# Anticoagulants and Statins As Pharmacological Agents in Free Flap Surgery: Current Rationale

**Published:** 2015-11-20

**Authors:** Adnan Pršić, Elizabeth Kiwanuka, Stephanie A. Caterson, Edward J. Caterson

**Affiliations:** ^a^Department of Plastic Surgery, Rhode Island Hospital, Brown University School of Medicine, Providence; ^b^the Division of Plastic Surgery, Brigham and Women's Hospital, Harvard Medical School, Boston, Mass

**Keywords:** free flap, microsurgery, statins, anticoagulants pharmacology

## Abstract

Microvascular free flaps are key components of reconstructive surgery, but despite their common use and usual reliability, flap failures still occur. Many pharmacological agents have been utilized to minimize risk of flap failure caused by thrombosis. However, the challenge of most antithrombotic therapy lies in providing patients with optimal antithrombotic prophylaxis without adverse bleeding effects. There is a limited but growing body of evidence suggesting that the vasoprotective and anti-inflammatory actions of statins can be beneficial for free flap survival. By inhibiting mevalonic acid, the downstream effects of statins include reduction of inflammation, reduced thrombogenicity, and improved vasodilation. This review provides a summary of the pathophysiology of thrombus formation and the current evidence of anticoagulation practices with aspirin, heparin, and dextran. In addition, the potential benefits of statins in the perioperative management of free flaps are highlighted.

Free flaps have become a common technique to transfer tissue, such as skin, fat, fascia, muscle, and/or bone, together with its blood supply. Microvascular free-tissue transfers are often used in an attempt to restore complex congenital, ablative, and traumatic tissue defects, and the survival of the flap is highly dependent on the patency of the microvascular anastomosis.^1^^,^^2^ During surgery, the arterial and venous blood flow is reconnected using microsurgical techniques. These techniques are based on standard vascular surgery principles, on a microscopic level. Murphy^3^ performed the first successful vascular anastomosis as early as 1897. In 1902, Carrel^4^ described the method for triangulation of blood vessels in arterial and venous repair, and in 1960, Jacobson and Suarez^5^ created microsurgical anastomoses in laboratory animals, coining the term “microvascular surgery.” Reconstructive microsurgery was born in the garage of Dr Harry Buncke in 1964, when he reported a successful replantation of a rabbit ear with 1-mm vessels.^6^ Buncke, and his homemade instruments, unlocked the door to modern-day microsurgical techniques. Initially, free flap surgery was restricted by high failure rates, long operative times, and a limited number of trained surgeons, but advances in surgical techniques and equipment have decreased the rates of complications. However, despite the current reliability of microsurgical free-tissue transfers, flap failures still arise. Such failures are most commonly related to thrombosis occurring at the arterial or venous anastomosis, often resulting from technical errors. Approximately 10% of microvascular free flaps suffer complications, and flaps that undergo reexploration can be salvaged at a 50% to 80% success rate, making the overall success rate for free flaps up to 98% among experienced surgeons.^7^^-^9 Nonetheless, anastomotic thrombosis still remains a major complication of microsurgical free-tissue transfers and total flap loss due to thrombi remains a significant concern.^10^

To inhibit the formation of microvascular thrombi, many surgeons routinely use preemptive anticoagulants and/or vasodilators.^1^^,^^11^^,^^12^ Approximately 96% of surgeons reported use of some types of anticoagulation regimen, and the protocols vary widely among surgeons.^13^ The most commonly used pharmacological agents are aspirin, heparin, and dextran, but their usage is often based on the surgeon's experience and without agreement in the timing or dosing. In fact, an early review of anticoagulation practices around the world point to anecdotal experience with more than 20 different agents used, most with very limited data supporting their benefit.^14^ Although some of the pharmacological agents have been studied experimentally, most of the data come from animal studies or retrospective studies, with only a few prospective randomized trials. In addition, new drug such as statins have emerged as pharmacological agents that are used to prevent the formation of microvascular thrombosis. There is a growing body of evidence that suggest that the vasoprotective and anti-inflammatory actions of statins can be beneficial for free flap survival, but their role in preventing anastomotic thrombosis is not known. This article provides a summary of the pathophysiology of thrombus formation and reviews the current evidence of anticoagulation practices with aspirin, heparin, and dextran. We revisit the literature concerning the utility of pharmacological treatments used to improve free flap survival and review the utility of pharmacological agents not traditionally thought of as anticoagulants.

## PATHOPHYSIOLOGY OF THROMBOSIS

The formation of thrombi is the body's defense mechanism to prevent blood loss after injury. Thrombosis is highly regulated through a complex interplay between the vascular endothelium, platelets, various molecular receptor, and inflammatory mediators. The microvascular anastomosis injures the vascular intima, causing the body to employ platelets and fibrin to seal the vascular defect. The time course of the development of a flow-occlusive thrombus is dependent on a multitude of factors, and the literature reveals that little is known about the underlying mechanisms. Limited evidence points out that 90% of arterial thrombi and 42% of venous thrombi in free flaps occur on postoperative day 1. The risk for anastomotic thrombosis is highest during the first 2 postoperative days and decreases to 10% after postoperative day 3.^11^^,^^12^^,^^15^ In agreement with these clinical observations, Chien et al^16^ showed that 95% of flap reexplorations were made during the first 72-hour postoperative period. Better knowledge of the mechanism and time course of thrombus formation in venous and arterial anastomoses is essential in directing the appropriate perioperative pharmacological treatment as a means to reduce ischemic tissue injury and ultimately flap failure.

Studies have shown that the content of microvascular thrombi differs between venous and arterial occlusions, making the choice of antithrombotic agent particularly important.^17^ Platelet aggregation is believed to be the underlying cause of arterial thrombosis, whereas venous thrombosis is primarily the result of fibrin clotting. In microvascular tissue transfer, venous thrombosis causes flap failure more frequently than arterial thrombosis, making fibrin depositions a more significant factor. Khouri et al^17^ showed that microvascular venous thrombi contained more fibrin than platelets and theoretically identified heparin as the better anticoagulant by selectively inhibiting platelet aggregation and fibrin deposition. Although other reports support the finding of Khouri et al, they also detected a higher content of platelets in arterial thrombi, making aspirin the preferred agent.^18^ However, on the basis of the finding of equal content of both fibrin strands and platelet aggregates in thrombi on electron microscopy, Savoie et al^19^ suggested the use of both aspirin and heparin in the prevention of anastomotic thrombi.

The difference in composition between arterial and venous thrombi can be attributed to the mechanisms of primary and secondary hemostasis. Primary hemostasis is initiated upon endothelial damage, exposing the normally isolated subendothelial collagen to circulating platelets. Platelets initially aggregate using the specific glycoprotein receptor Ia/IIa, and the adhesion is further strengthened by von Willebrand factor (vWF). Activated platelets release granules containing ADP, platelet-activating factor, serotonin, platelet factor 2, vWF, and thromboxane A_2_ (TXA_2_), which, in turn, activate additional platelets, increasing their binding to fibrinogen via glycoprotein IIb/IIIa. Secondary hemostasis occurs simultaneously and utilizes activated platelets and tissue factor (TF) to initiate the coagulation cascade. Secondary hemostasis results in the formation of fibrin strands and is believed to propagate the formation of venous thrombi.^20^^,^^21^ However, not mutually exclusive, the 2 processes are linked via multiple factors and seem to affect vascular patency through the formation of fibrin strands and platelet plugs.

## ANTICOAGULANTS IN FREE FLAP SURGERY

The addition of pharmacological anticoagulants in patients undergoing microvascular free-tissue transfers has become a mainstay of perioperative management (Table 1). Clinical observations have suggested that antithrombotic therapy significantly reduces the morbidity after microsurgical free-tissue transfers.^22^ It was proposed as early as 1978 by Ketchum^23^ that the outcome of free flap transfer could be improved by the addition of agents that decreased platelet function, increase blood flow or decrease blood viscosity, and counteract the effects of thrombin on platelets and fibrinogen. Chen et al^24^^,^^25^ have described the ideal antithrombotic agent as an easily administered anticoagulant that successfully prevents thrombosis, with minimal side effects and low rates of complications. However, to date, none of the available pharmaceutical agents have successfully met all the criteria listed previously.

### Aspirin

Aspirin is a potent anticoagulant used to decrease graft or vascular occlusion for a range of vascular procedures. Aspirin acetylates and thereby irreversibly inhibits cyclooxygenase (COX), which is the enzyme required for the formation of both prostaglandins and TXA_2_. Prostaglandins are potent vasodilators that inhibit platelet aggregation, whereas TXA_2_ both vasoconstricts and enhances platelet aggregation. Aspirin is rapidly absorbed in the upper gastrointestinal (GI) tract, and one of aspirin's major advantages is that it inhibits platelet function within 60 minutes of administration. In addition, the irreversible inhibition of COX results in decreased platelet aggregation for up to 10 days, which approximates the normal life span of a platelet.

Despite a relatively favorable safety profile, aspirin usage can be associated with various complications. Data from coronary artery bypass graft surgery show increased intraoperative blood loss and transfusion requirements with preoperative use of aspirin.^26^ In addition, inhibition of COX leads to lower levels of prostaglandin E_2_ (PGE_2_) and the subsequent loss of the protective effects of PGE_2_ on the gastric mucosa leads to the GI side effects associated with aspirin use. Specifically, aspirin use has been shown to increase the risk of GI bleeding, even in low doses.^27^ Other rare, but serious, complications attributed to aspirin usage are hemorrhagic stroke and increased sensitivity resulting in urticaria, rhinitis, and bronchoconstriction.

With regard to the prevention of thrombosis, no associated dose response has been shown for aspirin but higher doses increase the rate of GI side effects. Although studies in healthy patients and those with atherosclerotic disease have shown that 100 mg of aspirin effectively inhibits TXA_2_, previously reported anticoagulation protocols in microvascular surgery cite doses ranging from 250 mg every 3 days to as high as 1500 mg daily. However, low-dose aspirin (5 mg/kg) administered via intra-arterial infusion in an arterial and venous anastomoses rat model has been shown to successfully reduce thrombus formation and maintain the patency of the microcirculation. The benefit of low-dose aspirin is that it does not affect the COX of endothelial or smooth muscle cells, thereby leading fewer systemic side effects.^28^^,^^29^

There are no prospective randomized trials evaluating the benefit of aspirin administration in microvascular tissue transfer. In a retrospective chart study, Chien et al^16^ found that flap survival rates in patients undergoing head and neck reconstruction using coadministrating aspirin with subcutaneous heparin were equivalent to the free flap survival rates in patients using other anticoagulation agents. Basile et al^30^ showed that ticlopidine, an ADP receptor inhibitor, administered alone or in combination with aspirin, significantly increased the 1-hour patency rate in an animal thrombosis model. Unfortunately, the clinical utility of the combination treatment was never established. Therefore, recommendations for the use of aspirin in microvascular surgery are primarily based on animal models and on observations in cardiac and vascular surgery practices where a protective effect of aspirin against postoperative death, stroke, myocardial infarction, and vascular occlusion was shown. In addition, a preventive effect of aspirin on the formation of microvascular venous or arterial thrombosis in free-tissue transfers has not yet been shown, putting the use of aspirin in free flap surgery in question.

### Heparin and low-molecular-weight heparin

Heparin is a glycosaminoglycan, clinically used in the prevention of both arterial and venous thromboses.^31^ Heparin, and its low-molecular derivatives, binds to antithrombin, thereby causing a conformational change that increases the affinity of antithrombin to its substrate by a 1000-fold. Activated antithrombin inactivates coagulation factors II (thrombin), IX, X, XI, and XII, resulting in decreased thrombosis formation. In addition, inactivation of thrombin by antithrombin leads to reduced levels of coagulation factors V and VII, decreased recruitment of platelets, and reduced cross-linking of fibrin. The antithrombotic effect of heparin is clinically measured by an increase in clotting time and expressed as prolonged activated partial thromboplastin time (aPTT). When administered in large doses, heparin stimulates endothelial cells to produce nitric oxide (NO), leading to increased vasodilation. Low-molecular-weight heparin (LMWH) is a derivative of heparin with the same therapeutic effect on the inhibition of factor X, giving LMWH the same efficacy as heparin in preventing venous thrombosis. Because of its weaker inhibition of antithrombin, LMWH has fewer adverse effects than heparin. Heparin and LMWH are often considered standard of care in deep venous thromboembolism prophylaxis. However, because of conflicting reports in the literature, there is still debate whether LMWH is as efficient as heparin in preventing arterial thrombosis. Although some studies have clearly shown that LMWH is less effective than heparin in reducing the incidence of arterial thrombosis, other studies have reported equal results. Studies comparing the effects of heparin and LMWH in a rat model showed that both agents caused a similar increase in vessel patency of up to 50% compared with untreated controls.^32^ Other studies yielded similar results showing equal increase in free flap survival with the usage of heparin and LMWH, with the only notable difference between the 2 agents being a higher rate of hematoma development in the heparin group.^33^ In a rat arterial model, Malm et al^34^ addressed the specific concern of the ability of LMWH to reduce the formation of arterial thrombi. LMWH was shown to prevented arterial thrombosis, as effectively as heparin, but in contrast to heparin, LMWH did not increase the incidence of bleeding.

One potential goal of anticoagulation therapy in free-tissue transfer is to deliver the anticoagulant agent locally to the anastomotic site, thereby maintaining low systemic concentrations to reducing adverse effects. In animal studies, irrigation with a 100 U/mL solution of heparin has been shown to increase the patency of microvascular anastomoses at 24 hours, without a prolongation in aPTT.^35^ Fu and colleagues^36^ showed a significant increased patency rate of vascular anastomoses after local administration of high concentrations of heparin. Similarly, irrigation with heparin was shown to have a significant effect on the reduction of thrombosis during first 7 days after surgery as compared with saline irrigation alone and there was no observed difference in patency or side effects between heparin and LMWH.^25^ In a similar study, heparin was administered locally to musculocutaneous flaps after secondary venous ischemia and revascularization and flaps were randomized to treatment with low-dose systemic heparin, local infusion, or untreated control. Local heparin delivery resulted in complete survival of all flaps, whereas the control and systemic heparin groups had flap failures of 60.8% and 62.1%, respectively.^37^ Topical application also seems to have an effect on cutaneous free flaps. Sawada et al^38^ found that dorsal ischemic rat flaps treated with topical heparin had an increase in flap survival compared with areas not treated with heparin. Although these results are promising, no human studies have shown favorable results with topical heparin or LMWH treatment.

### Dextrans

Dextrans are a group of complex polysaccharides, with molecular weights ranging from 3 to 2000 kDa. Dextrans are synthesized by fermentation of sucrose by *Leuconostoc mesenteroides*, and their discovery dates back to the mid-19th century when Louis Pasteur^39^ described dextran as a microbial product in wine. Dextrans are now commonly used to decrease vascular thrombosis. The antithrombotic effects of dextrans are mediated by several different mechanisms. Dextrans bind to erythrocytes, platelets, and endothelial cells, thereby increasing their electronegativity and reducing aggregation and adhesion. Dextrans also inhibit α-2 antiplasmin, thereby promoting thrombolysis.^40^ In addition, the larger dextrans are excreted poorly from the kidney and the osmotic effects cause hemodilution with improved blood flow that further increases the patency of the anastomosis.^41^

The administration of dextrans varies globally, and there is no controlled randomized trial available to support their recommended use or confirm a beneficial effect in free flap survival.^42^ Although there are relatively few side effects associated with dextran use, when adverse effects do occur, they can be very serious. These include anaphylactic reaction, nephrotoxicity, pulmonary edema, and adult respiratory distress syndrome. Several institutions have found the rates of pulmonary edema to outweigh the benefits of dextrans, consequently limiting their use in free flap surgery and in patients awaiting angioplasty.^43^^,^^44^ Given the added risk of serious side effects and a lack of data proving the efficacy of dextrans to reduce free flap failure, many practice habits back that of other institutions and argue for cautious use of dextrans in free-tissue transfers.

## STATINS AS PHARMACOLOGICAL AGENTS IN FREE FLAP SURGERY

**Figure 1:**
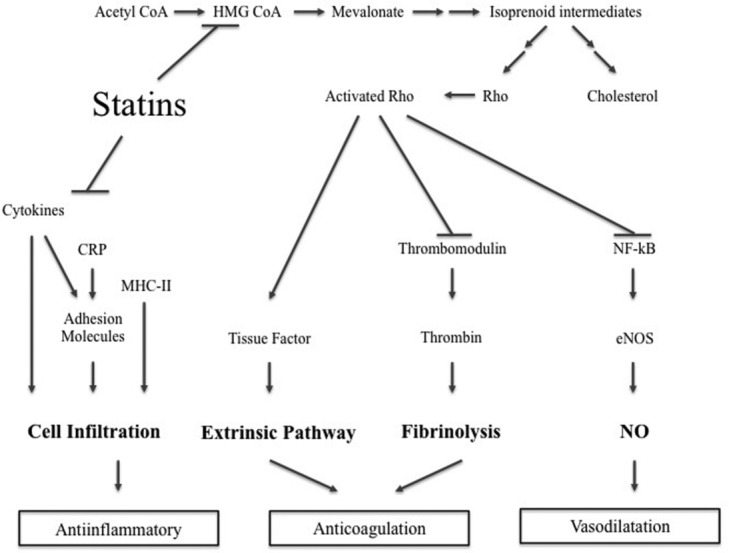
Overview of the mechanism of action. Inhibition of HMG CoA modulates cell infiltrate, decreases coagulation and promotes vasodilation. CoA indicates coenzyme A; CRP, C-reactive protein; HMG-CoA, 3-hydroxy-methylglutaryl coenzyme A; MHC-II, major histocompatibility complex class II; NF-κB, nuclear factors kappa beta; NO, nitric oxide; and eNOS, endothelial NO synthase.

3-Hydroxy-methylglutaryl coenzyme A (HMG-CoA) reductase inhibitors, otherwise known as statins, are powerful inhibitors of cholesterol biosynthesis (Fig 1). Statins are routinely used in the management of hyperlipidemia and have as such been established as a major agent in the prevention of coronary artery disease, stroke, and ischemic events.^45^^-^47 Statins inhibit the conversion of HMG-CoA to 1-mevalonic acid, the rate-limiting step of cholesterol synthesis. The inhibition of HMG-CoA reductase also leads to decreased formation of several intermediates that are important in the regulation of endothelial cell function.^48^^,^^49^ The inhibition of mevalonic acid synthesis prevents the synthesis of isoprenoid intermediates of the cholesterol biosynthetic pathway.^50^ These intermediates are important for the posttranslational modification of various proteins, including the γ-subunit of heterotrimeric G-proteins and small guanosine triphosphate–binding proteins such as Rho.^51^ Different statins have varying tissue permeability and metabolism, resulting in different potencies for extrahepatic HMG-CoA reductase inhibition. The more lipophilic statins, such as atorvastatin and simvastatin, enter the endothelial cells by passive diffusion, whereas the hydrophobic statins, such as pravastatin and rosuvastatin, primarily target the liver.

The endothelium maintains vascular homeostasis by controlling inflammation and coagulation, as well as regulating the vascular tone through the production of vasoactive agents. During free flap surgery, the function of the endothelium can be altered. The resulting endothelial dysfunction contributes to the formation of thrombi by inducing an inflammatory state that is marked by a reduction of anticoagulant factors and an increase in prothrombotic factors.^52^^,^^53^ Although it is known that a decrease in low-density lipoprotein (LDL) contributes to an improvement of endothelial dysfunction, in vivo studies have shown that statins restore endothelial function in patients with hyperlipidemia and atherosclerosis before decreasing lipid levels.^54^ These findings suggest additional mechanisms are involved in the therapeutic effect of statins on the vascular endothelium.^55^^-^58 In addition, statins have been shown to directly affect endothelial cell function and in vivo studies in a mouse model have shown that satins can modify vessel relaxation.^59^ Statins have been shown to exert their anticoagulative activities through several mechanisms that target inflammation, coagulation, and vasoconstriction.

The majority of clinical data regarding inflammation and statins come from large cohort studies in populations with hyperlipidemia and associated vascular and cardiovascular diseases. Results of clinical trials such as CARE, LIPID, and HPS suggested that the cardiovascular benefit of statins in part correlated only with the reduction of LDL and went beyond the lipid-lowering properties, pointing to alternate pathways of action. Over the years, culminating evidence has shown that one of the major effects of statins includes the reversal of endothelial dysfunction exerted by the anti-inflammatory properties of statin.^49^ A major component of the anti-inflammatory mechanism is the direct inhibition of intracellular adhesion molecule-1 (ICAM-1) and vascular cell adhesion molecule-1 (VCAM-1).^60^^,^^61^ The proinflammatory cytokines that are produced upon endothelial injury facilitate leukocyte migration across the endothelium by increasing the expression of both ICAM-1 and VCAM-1.^62^ The local inflammatory milieu is further established by the production of the inflammatory cytokines IL-1, IL-6, tumor necrosis factor-alpha (TNF-α), and transforming growth factor-beta (TGF-β) by leukocytes. Statins have been shown to downregulate the adhesion molecules ICAM-1 and VCAM-1, thereby leading to decreased neutrophil rolling, adherence, and migration across the endothelium.^63^^,^^64^ Moreover, statins have been shown to inhibit the expression of major histocompatibility complex class II (MHC-II) on endothelial cells and macrophages, thereby repressing T-cell activation.^65^

In addition to decreasing the inflammatory response, current data indicate that statins can modulate the levels of the highly sensitive C-reactive protein (CRP).^53^ While previously thought of as an acute-phase reactant in inflammatory processes, recent studies have shown that CRP can upregulate the expression of adhesion molecules and chemokines in endothelial cells and act like a mediator of thrombosis following vascular injury.^66^^,^^67^ Statins have been shown to reduce CRP levels independent of the decrease in serum lipid concentration.^68^ It has been confirmed by multiple prospective cohort studies that CRP levels are useful predictors of myocardial infarction and stroke and serve as important indicators of vascular and cardiovascular health.^69^

C-reactive protein has been identified as a leading biomarker of inflammation in clinical setting and a strong independent predictor of future vascular events.^70^^-^73 It has been suggested that the decreased levels of CRP seen with the use of statins mediate the beneficial impact on the reduction of coronary events and the improved survival after acute coronary syndrome.^74^^,^^75^ Data from the most recent JUPITER trial (Justification for the Use of Statins in Prevention: an Intervention Trial Evaluating Rosuvastatin) showed a reduction in all vascular events by 44%, a 54% reduction in the rate of myocardial infarction and 48% reduction in stroke, in a cohort of 17,802 patients with normal serum LDL levels (<130 mg/dL) and elevated CRP levels (≥2.0 mg/L) treated with rosuvastatin versus placebo.^47^ These findings support the anti-inflammatory effects of statins on the protection of vascular health independent of lipid lowering.

The prothrombotic state that follows endothelial dysfunction is characterized by an activation of the extrinsic coagulation pathway, triggered by the release of TF. Coagulation is further promoted by the inhibition of thrombolysis mediated by the increased levels of plasminogen activator inhibitor-1 (PAF-1). Tissue factor is a glycoprotein that converts factor X to factor Xa and is the principal initiator of the extrinsic coagulation pathway; TF can be found in the subendothelial layers of the vessel wall. In addition, substantial amounts of TF can be released by macrophages and monocytes after stimulation with inflammatory cytokines.^76^ Upon injury of the vessel wall, the concentration of TF increases, thereby initiating the coagulation cascade causing the formation of a thrombus. In vitro studies have confirmed that the prevention of isoprenoid intermediate synthesis by statins is crucial for the inhibition of TF. Although statins have traditionally not been used as anti-TF agents, decreased expression of TF mRNA has been seen in macrophages, monocytes, and aortic endothelial cells after the use of statins.^77^^-^79 Studies in in vivo atherosclerotic animal models have shown a reduced TF expression with statin treatment that is independent of lipid lowering.^80^^-^82 In addition to the results obtained in animal studies, the ATROCAP (Atorvastatin and Thrombogenicity of the Carotid Atherosclerotic Plaque) study has shown lower TF antigen levels and TF activity with low-dose atorvastatin compared with placebo, pointing to a clinical utility of statins in reduction of TF and initiation of the coagulation cascade.^83^ The inhibition of isoprenoid intermediates also explains the upregulation of thrombomodulin (TM) by statins. These intermediates exert anticoagulant properties by activating a cofactor in thrombin-induced activation of the protein C pathway.^84^ Statins have been shown to increase TM mRNA levels in a dose-dependent manner using the Rho signaling pathway.^85^

The endothelium promotes vasodilation by the production of NO by means of endothelial NO synthase (eNOS). eNOS is the molecular factory of NO and its stability and activity directly regulate the production of NO, a potent vasodilator and inhibitor of platelet aggregation.^86^^-^88 Early landmark studies by Laufs et al^89^ demonstrated the ability of statins to upregulate eNOS activity and increase NO production both under normal conditions and after hypoxia. Through their inhibition of the mevalonate pathway, statins inhibit isoprenoid production, which leads to altered posttranslational regulation of the Rho signaling pathway.^35^ Rho modulates the activity of nuclear factors kappa beta (NF-κB) and Kruppel-Like factor-2 (KLF2) and inhibits endothelial production of eNOS. By inhibiting the isoprenylation of Rho and Rho kinase, statins increased eNOS expression, eNOS mRNA stability, and thereby NO production.^58^,^89^^-^92 Statins can increase eNOA activity by posttranslational activation of the phosphatidylinositol 3-kinase/protein kinase Akt pathway.^93^ Activation of Akt is an important event in many cellular activities and Akt can regulate eNOS activity, leading to increased NO production.^94^

Rat burn models have also been used as a means to study the effects of statins on the endothelium. Zones of stasis, thought to develop from resulting heat injury to the endothelium, were used by Uygur et al^95^ as a model to display the reduction in the vasoactive and antithrombotic properties of the endothelium. Their model showed an impressive ability of simvastatin to salvage zones of stasis. Published data showed statistically significant difference in blood flow favoring simvastatin versus control group in burn interspaces, whereas TM immunohistochemistry staining showed preservation of endothelial TM only in the simvastatin group.^95^ Gross appearance of the tissue also displayed increased viability in the simvastatin group compared with controls. On the basis of such experiments and the culmination of additional evidence on statins, a mechanism for tissue preservation might stem from the statin-induced increase in vasoreactivtiy secondary to increased eNOS expression/RNA stability, NO production, and a simultaneously decreased rate of thrombosis secondary to the upregulation/preservation of TM. Equally relevant in statin-induced anticoagulation were findings by Undas et al,^96^ who observed statistically significant reduction of prothrombin activation products, factor Va and factor XIIIa, with simvastatin use over a course of 3 months in patients with advanced coronary artery disease. The evidence here points to a decreased activation in blood clotting with simvastatin independent of cholesterol reduction.

In agreement with above-referenced studies, clinical studies in patients with atherosclerosis have shown statistically significant effects of therapeutic doses of atorvastatin on Rho kinase inhibition whereas additional studies with the Rho inhibitor fasudil have yielded statistically significant effects showing improved vasodilatation, as measured by endothelium-dependent, flow-mediated brachial artery ultrasonography.^97^^,^^98^ These observations confirm the findings of Laufs et al and add to the suggestion that inhibition of Rho/Rho kinase with atorvastatin has the potential to contribute to an increase in baseline eNOS activity and NO availability in clinical use outside of cell culture models.

The pathophysiology behind ischemic and ischemia-reperfusion (I/R) injuries seen in solid organ transplantation is similar to that of free-tissue transfers and equally leads to increased inflammation, coagulation, and vasoconstriction associated with endothelial dysfunction.^99^^,^^100^ Of relevance to free-tissue transfers are the I/R injury in vivo animal models of myocardial ischemia, showing short-term cardioprotective effects of statins.^101^^,^^102^ This was attributed to the ability of statins to preserve the activation of eNOS immediately after I/R injury. To further confirm the lipid independent effects of statins on I/R injury, studies in normocholesterolemic rats have shown that statin pretreatment 18 hours prior to induction of I/R injury has a protective effect on the myocardium while improving coronary flow.^103^^,^^104^ Animal studies in I/R injuries have identified TNF-α as a key mediator in endothelial dysfunction, and by decreasing the production of NO via the NF-κB pathway and stimulating the production of oxygen free radicals, TNF-α further adds to the release of inflammatory cytokines.^105^ This creates the same proinflammatory milieu thought to contribute to endothelial dysfunction in atherosclerosis.

## DISCUSSION

Microvascular free-tissue transfers are key procedures in reconstructive surgery. Although the success rates for free flaps are increasing, studies have identified venous thrombosis, and the resulting venous congestion, as the most common reason for flap failure. The thrombotic events are often triggered by suboptimal microvascular anastomosis, pedicle compression, infection, or donor-vessel mismatch. Several pharmacological agents have been used in an attempt to improve flap survival, and agents such as aspirin, heparin, and dextran are often added preoperatively in an attempt to prevent the formation of thrombi. However, there are limited clinical studies supporting their use in free flaps and there is currently no consensus for an ideal regimen. No data clearly support the use of aspirin, heparin, or dextran in perioperative anticoagulation in microsurgical free-tissue transfers. Current methods of anticoagulation in microvascular surgery have not been found to be consistent in their efficacy and profile of adverse effects. Specifically, aspirin, heparin, and dextran have all varied greatly in their results in animal trials compared with prospective and retrospective clinical trials in humans. In addition, the pleiotropic effects of statins have raised the question whether statins could be beneficial in the prevention of microvascular thrombosis after free-tissue transfers.^49^^,^^106^^,^^107^

Since the approval of the first HMG-CoA reductase inhibitor lovastatin in 1987, statins have been prescribed to more than 1 million patients and are generally considered to be safe and efficacious pharmaceutical agents. By inhibiting mevalonic acid, the downstream effects of statins include reduction of inflammation, reduced thrombogenicity, and improved vasodilation. However, over the course of the last 2 decades, side effects, particularly rhabdomyolysis and adverse effects on liver, were noted in several trials. The incidence of myalgia reported in the literature is said to occur in about 5% of cases, whereas true cases of rhabdomyolysis range from 0.1% to up to 0.2% combined with side effects of additional pharmaceutical agents. Equally prevalent is the notion that statins cause adverse effects on the liver. Most reports of liver failure were reported in isolated case reports. However, a large trial following 23,000 patients on statins noted a significant increase in alanine aminotransferase on only 0.3% of the enrolled patients, disputing the idea that statins caused severe injury to the liver.^108^ In comparison with aspirin, heparin, and dextran, statins have not been shown to pose any increased risk of bleeding or impairment of liver function. In addition, other major adverse effects, such as heparin-induced thrombocytopenia, pulmonary edema, or nephrotoxicity, as seen with heparin or dextran, are not a part of the risk profile.

Given the fact that improvement in endothelial dysfunction is seen prior to and independent of changes in lipid concentrations, we postulate that statin therapy has the potential to increase endothelial NO bioavailability in zones of mechanical or ischemic injury in the microcirculation, leading to vasodilatation and further reduction in microvascular thrombosis. Not only is this effect theoretically possible in free-tissue transfers but also in the microvasculature of local and regional tissue rearrangements.

There is a limited body of evidence supporting the use of statins as anticoagulants in free flap surgery. In a review of the surgical literature, Williams and Harken^109^ conclude that postoperative mortality could be reduced with statin use. A well-designed prospective randomized study with atorvastatin in elective vascular surgery yielded results that showed an event incidence rate marked by nonfatal myocardial infarction, stroke, and death of 3 times higher for patients treated with placebo versus patients treated with atorvastatin.^110^ An often-cited meta-analysis by Hindler et al^111^ states that both cardiac and general surgery patients show better clinical outcomes with the addition of statins. Despite a limited body of evidence, Karsenti and colleagues^107^ have proposed and instituted a preoperative 2-week treatment protocol with statins, adding 40 mg of atorvastatin per day to all free flap surgical procedures. In addition, the administration of atorvastatin is continued through the postoperative period until complete stabilization of the patient.^107^ Although the data are promising, we argue that a better understanding of the molecular pathways and expression of molecular factors involved in vasoreactivtiy, thrombosis, and inflammation in free flap surgery is needed before starting empirical treatment with statins. A start with animal models to assess the effects of statins on the site of vascular anastomosis and to study the effects on ischemia and injury should be attempted prior to any large trails or empirical treatment. An equally important aspect of statin treatment is the cost-effectiveness of such therapy. Well-designed studies marked by a primary endpoint of free flap survival with statin treatment are also needed to determine the number needed to treat and the cost-effectiveness of statin treatment in free flap surgery.

## CONCLUSION

The trend toward more elective microsurgery, in combination with the clear molecular pathway behind the effects of satins, suggests that placing patients with elective free flap taking statins as a part of their normal perioperative routine may be advantageous. With low complication rates, statins offer a relatively benign intervention that may significantly benefit microsurgeons and patients alike. Studies need to be conducted to determine the optimal pre- and postoperative treatment length and dose of statin therapy.

## Figures and Tables

**Table 1:** Overview of the mechanism of action of the most commonly used anticoagulant in free flap surgery

Drug	Mechanism of action	Levels of evidence in free flap surgery
Aspirin	Inhibits the enzyme cyclooxygenase, thereby preventing the formation of both thromboxane and prostacyclin	Level IV
Heparin	Bind to antithrombin and increases the affinity of antithrombin to its substrate by a 1000-fold. Activated antithrombin inactivates coagulation factors II (thrombin), IX, X, XI, and XII resulting in decreased thrombosis formation.	Level IV
Dextran	Bind to erythrocytes, platelets, and endothelial cells and increase their electronegativity and reduce aggregation and adhesion. Dextrans also inhibit α-2 antiplasmin, thereby promoting thrombolysis. The osmotic effects cause hemodilution with improved blood flow.	Level IV
Statins	Reduces inflammation, reduces thrombogenicity, and improves vasodilation.	Level IV
